# Effect of a Low-Molecular-Weight Allosteric Agonist of the Thyroid-Stimulating Hormone Receptor on Basal and Thyroliberin-Stimulated Activity of Thyroid System in Diabetic Rats

**DOI:** 10.3390/ijms26020703

**Published:** 2025-01-15

**Authors:** Kira V. Derkach, Alena S. Pechalnova, Viktor N. Sorokoumov, Inna I. Zorina, Irina Y. Morina, Elizaveta E. Chernenko, Egor A. Didenko, Irina V. Romanova, Alexander O. Shpakov

**Affiliations:** 1 Sechenov Institute of Evolutionary Physiology and Biochemistry, Russian Academy of Sciences, St. Petersburg 194223, Russia; derkatch_k@list.ru (K.V.D.); pechalnova.alena@gmail.com (A.S.P.); sorokoumov@gmail.com (V.N.S.); zorina.inna.spb@gmail.com (I.I.Z.); irinamorina@mail.ru (I.Y.M.); llystdi@gmail.com (E.E.C.); didenkoegor58@mail.ru (E.A.D.); irinaromanova@mail.ru (I.V.R.); 2 Institute of Chemistry, St. Petersburg State University, St. Petersburg 199034, Russia

**Keywords:** diabetes mellitus, hypothyroidism, thyroid hormone, thyroid-stimulating hormone receptor, allosteric agonist, thyroliberin

## Abstract

The approaches to correct thyroid deficiency include replacement therapy with thyroid hormones (THs), but such therapy causes a number of side effects. A possible alternative is thyroid-stimulating hormone (TSH) receptor activators, including allosteric agonists. The aim of this work was to study the effect of ethyl-2-(4-(4-(5-amino-6-(*tert*-butylcarbamoyl)-2-(methylthio)thieno[2,3-d]pyrimidin-4-yl)phenyl)-*1H*-1,2,3-triazol-1-yl) acetate (TPY3m), a TSH receptor allosteric agonist developed by us, on basal and thyroliberin (TRH)-stimulated TH levels and the hypothalamic-pituitary-thyroid (HPT) axis in male rats with high-fat diet/low-dose streptozotocin-induced type 2 diabetes mellitus (T2DM). Single and three-day administration of TPY3m (i.p., 20 mg/kg) was studied, and the effect of TPY3m on the HPT axis was compared with that of levothyroxine. TPY3m increased TH levels when administered to both healthy and diabetic rats, normalizing thyroxine and triiodothyronine levels in T2DM and, unlike levothyroxine, without negatively affecting TSH levels or the expression of hypothalamic and pituitary genes responsible for TSH production. TPY3m pretreatment preserved the stimulatory effects of TRH on TH levels and thyroid gene expression. This indicates the absence of competition between TPY3m and endogenous TSH for TSH receptor activation and is supported by our in vitro results on TPY3m- and TSH-stimulated adenylate cyclase activity in rat thyroid membranes. Morphological analysis of thyroid glands in diabetic rats after three-day TPY3m administration shows an increase in its functional activity without destructive changes. To summarize, TPY3m, with the activity of a partial allosteric agonist of the TSH receptor, was created as a prototype of drugs to correct thyroid insufficiency in T2DM.

## 1. Introduction

The hypothalamic-pituitary-thyroid (HPT) axis plays a key role in the regulation of metabolism and thermogenesis, as well as the functions of the cardiovascular, nervous, and musculoskeletal systems [1,2]. Changes in the activity of the components of this axis lead to various forms of thyroid pathology, which are characterized by both hypofunction of the thyroid gland (TG) and its hyperactivation. One of the common causes of this is autoantibodies produced by the thyroid-stimulating hormone (TSH) receptor and the components of the thyroid hormone (TH) synthesis system in thyrocytes, which leads to autoimmune diseases, such as autoimmune hyperthyroidism (Graves’ disease) and autoimmune thyroiditis (Hashimoto’s disease) [3,4]. Thyroid dysfunctions are often detected in patients with metabolic disorders, including type 2 diabetes mellitus (T2DM), and this is due to both metabolic and hormonal disturbances, as well as the activation of inflammatory, degenerative, and autoimmune processes [5,6,7]. Latent (subclinical) and manifest (clinical) hypothyroidism are most often associated with T2DM, which are diagnosed in 11.87 and 7.75% of diabetic patients, respectively [7]. The combination of T2DM and various forms of hypothyroidism is a risk factor for the development of diabetic nephropathy, diabetic retinopathy, a wide range of cardiovascular diseases, and diabetic neuropathy [8]. This indicates the significant importance of the pharmacological correction of T2DM-associated hypothyroidism, including restoring the activity of the signaling and effector components of TSH-dependent pathways in thyrocytes.

For the treatment of various forms of hypothyroidism, including Hashimoto’s disease, replacement therapy with TH-based drugs, most often levothyroxine, is used [9]. However, such therapy can lead to a number of serious side effects since TH-based drugs can cause heart rhythm disturbances and osteoporosis [10]. In addition, they negatively affect the activity of the HPT axis via a negative feedback mechanism, which, when they are withdrawn, leads to thyroid deficiency. In recent years, thyromimetics have been developed that selectively stimulate the β-isoform of nuclear TH receptors (β-TR), which is responsible for most of the “beneficial” effects of THs at the periphery and does not have a negative effect on musculoskeletal and cardiovascular systems [11,12,13]. The in vivo experiments show that β-TR-selective thyromimetics demonstrate high efficacy in subclinical hypothyroidism associated with obesity and dyslipidemia [14,15,16]. However, the selectivity of their action, although it allows for avoiding some side effects, including those caused by the negative impact of TH-based drugs on the cardiovascular system, also has a number of disadvantages and limitations, as a result of which none of the β-THR-selective thyromimetics have yet been approved for clinical use [12,15]. This indicates the relevance of developing new classes of thyroid stimulators, both in terms of their mechanism of action and chemical structure.

Of greatest interest are compounds that, under hypothyroidism, are capable of moderately stimulating TH production, maintaining the blood level of triiodothyronine (T3), the main effector hormone of the HPT axis, within the range of its reference values. This is important for maintaining the functional activity of the hypothalamic and pituitary links of the HPT axis. Possible candidates for such compounds are low-molecular-weight allosteric agonists of the TSH receptor, which target allosteric sites located in the transmembrane domain of the receptor [17,18,19,20]. It is important that allosteric regulators of GPCRs are characterized by high specificity for certain GPCR subtypes and are capable of selectively triggering certain intracellular signaling cascades, which creates an advantage of their use in comparison with orthosteric site ligands [20,21,22,23]. Along with this, they, as a rule, act on the GPCR-regulated effector system more moderately, do not prevent the functional interaction of endogenous orthosteric agonists with the receptor, and are able to restore the activity and trafficking of GPCRs with inactivating mutations, acting as pharmacological chaperones [20,22,24,25,26]. A significant advantage of allosteric GPCR regulators is the wide spectrum of their pharmacological activity since they can function as negative and positive modulators of signals generated by orthosteric ligands and can have their own activity or combine the properties of modulators and agonists [27,28,29,30].

We previously developed the compound TPY3m, a thieno[2,3-d]-pyrimidine derivative, with partial TSH receptor agonist properties [31,32]. This compound increased the expression of the genes involved in TH synthesis when incubated with FRTL-5 rat thyrocytes [31], and when administered intraperitoneally and orally to rats, it increased the blood levels of thyroxine (T4) and T3 while having minimal effect on blood TSH levels [32]. However, the efficacy of this compound for the correction of thyroid deficiency, including that caused by T2DM, has not been assessed. Its regulatory effects on various components of the HPT axis have not been studied. The effect of this compound on the stimulating effects of thyroliberin, the TSH-releasing factor (TRH), in animals with TH deficiency has also not been evaluated. It should be noted that, at present, such studies have not been carried out for any other low-molecular-weight allosteric regulators of the TSH receptor.

The aim of this work was to investigate the effect of TPY3m on basal and TRH-stimulated TH levels and the functional state of the HPT axis in male rats with T2DM induced by a long-term high-fat diet and a low-dose streptozotocin (STZ). Single and three-day intraperitoneal administration of TPY3m was studied, and in the case of three-day administration, the effect of TPY3m on the HPT axis was compared with that of levothyroxine, which is widely used as a replacement therapy for the treatment of hypothyroidism. The effect of three-day administration of this drug to rats on the morphology of thyroid tissue was also assessed. We found for the first time that TPY3m, a thieno[2,3-d]-pyrimidine derivative with properties of an allosteric partial agonist of the TSH receptor, can be used to compensate for thyroid deficiency caused by high-fat diet/STZ-induced T2DM without a significant inhibitory effect on TSH levels. It is important that TPY3m maintains TSH-induced TH production and, unlike levothyroxine, does not cause a decrease in the activity of the HPT axis. Based on the morphological data, it was shown that when administered to diabetic rats for three days, TPY3m increased the thyroid functional activity but did not induce destructive changes in TG. All this indicates that the compound TPY3m developed by us can become a prototype for drugs to correct T2DM-induced hypothyroidism and other thyroid-deficient pathology.

## 2. Results

### 2.1. Effect of TPY3m on Basal and TSH-Stimulated Adenylate Cyclase Activity in Thyroid Membranes

**Figure 1:**
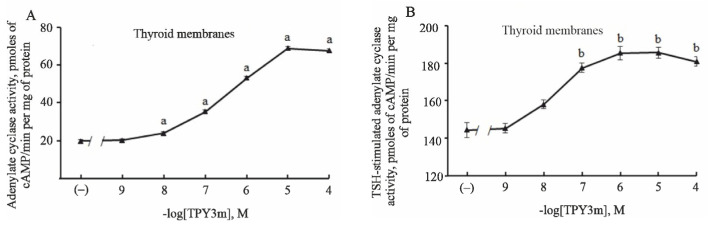
Effect of different concentrations of TPY3m on the basal (**A**) and TSH-stimulated (**B**) adenylate cyclase activity in the rat thyroid membranes. (**A**) The basal AC activity was 19.7 ± 0.8 pmol cAMP/min per mg of membrane protein. Stimulating effects of TPY3m were assessed in the concentration range from 10^−9^ to 10^−4^ M. (**B**) The AC activity stimulated by TSH (10^−9^ M) was 144.2 ± 4.1 pmol cAMP/min per mg of membrane protein (+632% over the basal AC activity). Combined action of TSH and TPY3m were assessed in the presence of 10^−9^–10^−4^ M TPY3m. ^a^ The differences from the basal AC activity are significant at *p* < 0.05. ^b^ The differences from AC activity stimulated by TSH alone (in the absence of TPY3m) are significant at *p* < 0.05.

To assess the ability of TPY3m to activate the TSH receptor and trigger the AC signaling cascade in thyrocytes, we studied the effect of this compound on basal AC activity in membranes isolated from rat TG. At concentrations from 10^−8^ to 10^−4^ M, TPY3m increased cAMP production in a dose-dependent manner (Figure 1), and the EC_50_ value for its stimulatory effect was 329 ± 9 nM. After that, the effect of TPY3m on the AC activity stimulated by 10^−9^ M TSH was studied. According to the preliminary experiments, the AC-stimulating effect of TSH at this concentration is 75–80% of its maximum effect. With the combined action of TSH and low concentrations of TPY3m, not only was the additivity of their stimulating effects on cAMP production demonstrated but also the potentiation of the TSH effect in the presence of TPY3m. The increase in TSH-stimulated AC activity in the presence of 10^−7^ M TPY3m averaged 33.3 pmol cAMP/min per mg of membrane protein, which is twice the AC-stimulating effect of TPY3m alone at the same concentration. At higher concentrations of TPY3m, partial additivity of the stimulatory effects of TPY3m and TSH was observed (Figure 1). In the presence of 10^−4^ M TPY3m, the increase in AC activity above the TSH-stimulated enzyme activity was 25% less than that observed with the effect of TPY3m alone on basal AC activity.

In plasma membrane fractions isolated from the rat testes, AC activity upon the addition of TPY3m in the concentration range from 10^−8^ to 10^−4^ M did not differ from basal AC activity, indicating the absence of an effect of this compound on testicular AC (Figure S1). These membranes are enriched in luteinizing hormone (LH) receptors structurally close to the TSH receptor. The obtained data indicate the specificity of TPY3m action in relation to the TSH receptor and the absence of its effect on the activity of the LH receptor.

### 2.2. Effect of TPY3m on Basal and Thyroliberin-Stimulated Thyroid Hormones Levels in the Blood of Healthy Rats

**Figure 2:**
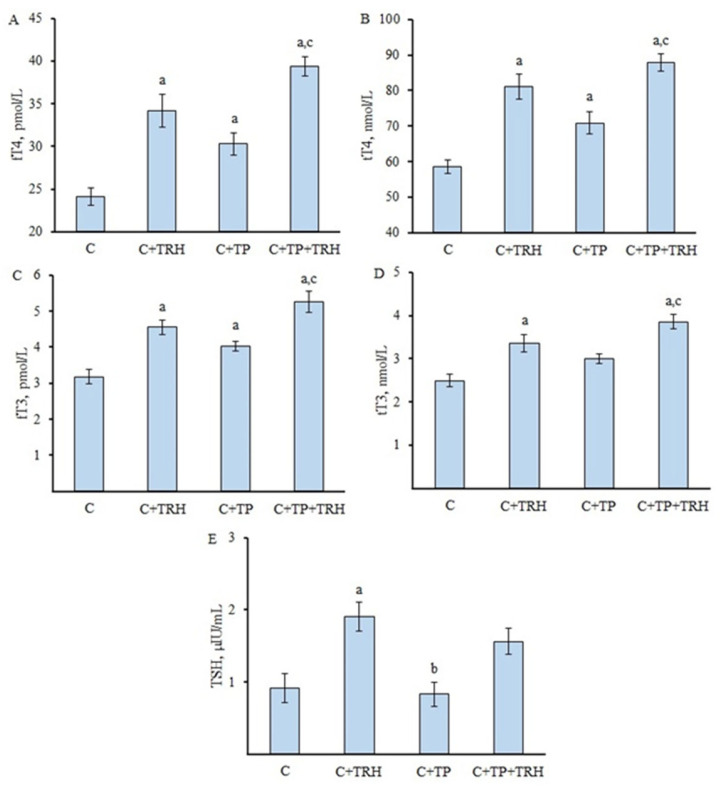
Effect of treatment with TPY3m (single dose 20 mg/kg, i.p.) on the basal and thyroliberin-stimulated levels of thyroid hormones and TSH in the blood of healthy rats. (**A**)—fT4, (**B**)—tT4, (**C**)—fT3, (**D**)—tT3, (**E**)—TSH. Differences with the control (^a^), C + TRH (^b^), and C + TP (^c^) groups are significant at *p* < 0.05. Data are presented as M ± SEM, and in all groups, *n* = 6.

At 3.5 h after the intraperitoneal administration of 20 mg/kg TPY3m to adult male rats, a significant increase in the blood levels of fT4, tT4, and fT3 was shown, and these effects were comparable to those of intranasally administered TRH, which exerts its effects through the activation of TSH release by pituitary thyrotrophs (Figure 2). The dose of TPY3m was chosen based on preliminary studies showing that this dose allows 70–75% of the maximal stimulatory effect of TPY3m on TH production to be achieved. When rats were pretreated with TPY3m, the stimulatory effect of TRH on TH levels was maintained, and a trend toward an increased effect of TRH was observed, although the differences between the C + TRH and C + TP + TRH groups were not significant (Figure 2). TRH caused a significant increase in the TSH level in the blood of animals, while TPY3m did not have such an effect. It was also shown that in the C + TP + TRH group, the TSH level did not differ significantly from that in the control (Figure 2). It has also been shown that TPY3m does not affect the blood testosterone level in male rats (Table S1).

### 2.3. Study of the Effect of TPY3m on Basal and Thyroliberin-Stimulated Expression of Thyroidogenic Genes in the Rat Thyroid Gland

The treatment of rats with TRH resulted in the stimulation of the expression of a number of genes responsible for TH synthesis. In the C + TRH group, the expression of genes encoding thyroglobulin (gene *Tg*), which is a source of tyrosine residues for TH synthesis; thyroid peroxidase (gene *Tpo*), responsible for the generation of hydrogen peroxide, which is necessary for iodination of tyrosine residues; sodium/iodide symporter (gene *Nis*), which ensures the transport of iodine to the thyrocyte compartments where TH are synthesized; and type 2 deiodinase (gene *Dio2*), catalyzing the conversion of thyroxine into the active form of T3, was increased (Table 1). TPY3m treatment resulted in an increased expression of *Tpo* and *Dio2* genes. The increase in *Dio2* gene expression was comparable to that in the C + TRH group, while in the case of the *Tpo* gene, the stimulatory effect of TPY3m was inferior to that of TRH (Table 1). For the combined action of TPY3m and TRH, the stimulating effect of TRH on the expression of all four genes involved in TH synthesis was preserved. No changes in the expression of the TSH receptor gene were detected in any of the studied groups (Table 1). Thus, in the TG of healthy rats, TPY3m stimulated the expression of genes responsible for TH synthesis (*Tpo* and *Dio2*) and ensured the maintenance of the stimulating effect of TRH on the expression of these and other (*Tg*, *Nis*) genes but did not affect the expression of the TSH receptor gene.

### 2.4. Metabolic and Hormonal Parameters, Including Thyroid Status, in Rats with Type 2 Diabetes Mellitus

T2DM was induced in rats by a long-term (16-week) high-fat diet and a low-dose STZ injection (10 weeks after the start of the diet, i.p., 25 mg/kg). Rats with T2DM showed increased body weight and elevated blood glucose, glycated hemoglobin, triglycerides, and total cholesterol levels, as well as impaired glucose tolerance, as indicated by a significant increase in glucose levels 120 min after glucose load in the IGTT (55% higher than in the control). Diabetic animals had increased blood insulin and leptin levels after a glucose load and increased basal leptin levels (Table 2). In diabetic animals, insulin resistance (IR) indices calculated both for the baseline state (fasting) and 120 min after the glucose load in the IGTT were significantly increased. In the latter case, the IR index was 238% higher than in control animals 120 min after the glucose load and 130% higher than in diabetic rats before the IGTT (Table 2). These data indicate the development of such characteristic signs of T2DM as obesity, hyperglycemia, impaired glucose tolerance, insulin resistance, hyperleptinemia, and dyslipidemia in rats fed a high-fat diet and injected with a low-dose STZ.

**Figure 3:**
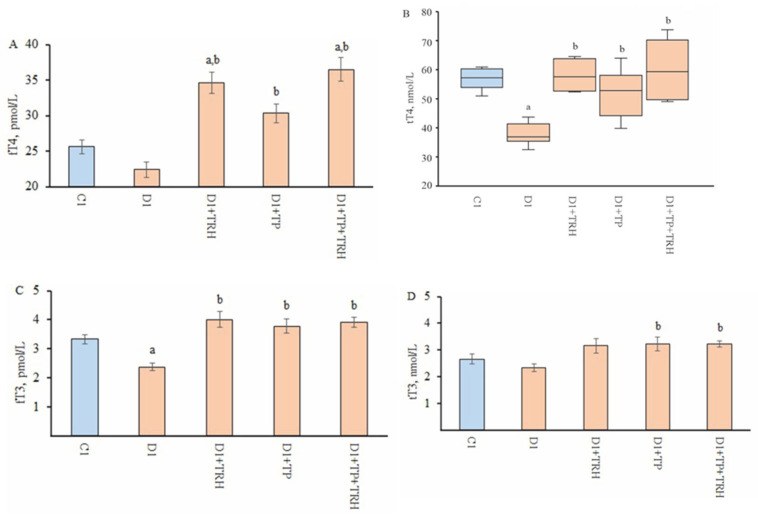
Effect of single-dose TPY3m administration (20 mg/kg, i.p.) on basal and thyroliberin-stimulated thyroid hormone levels in rats with high-fat diet/low-dose streptozotocin-induced T2DM. (**A**)—fT4, (**B**)—tT4, (**C**)—fT3, (**D**)—tT3. The differences with the C1 (^a^) and D1 (^b^) groups are significant at *p* < 0.05. The data on the blood tT4 levels are not normally distributed and are presented as median and interquartile ranges (25%; 75%). The data on the blood levels of fT4, fT3, and tT3 are normally distributed and are presented as M ± SEM. In all groups, *n* = 6.

The blood tT4 and fT3 levels in diabetic rats were reduced compared to control animals, while the blood fT4 and tT3 levels did not differ from the control, although they showed a tendency to decrease (Figure 3). These data indicate the development of TH deficiency in rats with long-term T2DM.

### 2.5. Effect of Single-Dose Administration of TPY3m on Basal and Thyroliberin-Stimulated Thyroid Hormones Levels and Thyroid Gene Expression in Diabetic Rats

TPY3m, like TRH, significantly increased blood TH levels when administered once to diabetic rats, but they did not exceed those in the control group (Table 3). In the D1 + TP + TRH group, with the combined action of the drugs, the stimulating effects of TRH were preserved, and the fT4 level in the D1 + TP + TRH group, as in the D1 + TRH group, was higher than in the C1 and D1 groups (Table 3). This indicates both the preservation of the thyrocyte response during their stimulation with TSH and TPY3m in rats with T2DM and the maintenance of their stimulating effects under these conditions when administered in combination.

In the group D1 + TP, compared with the D1 group, TPY3m increased the expression of *Tg* and *Nis* genes (Table 3). Unlike TRH, TPY3m did not significantly increase the expression of the *Tpo* gene and did not have an inhibitory effect on the expression of the TSH receptor gene, which was reduced in the D1 + TRH group. TPY3m increased the expression of the *Dio2* gene compared with the control but not with the D1 group (Table 3). In the case of the combined action of TRH and TPY3m in the TG of diabetic animals, an increase in the expression of all the studied genes involved in TH synthesis, with the exception of *Dio2*, was shown. For the *Nis* gene, an increase in its expression was demonstrated both in comparison with the control and diabetic groups and the group treated with TRH alone (Table 3). Similar to the D1 + TRH group, with the combined action of the drugs, the expression of the TSH receptor was decreased. In the D1 + TRH, D1 + TP, and D1 + TP + TRH groups, an increase in the expression of the type 3 deiodinase gene (*Dio3*), responsible for TH inactivation, was shown, but the differences were only significant between the D1 and D1 + TP + TRH groups (Table 3). Thus, the stimulating effects of TRH and TPY3m on TH production, including their combined action, were largely due to their ability to increase the expression of some genes responsible for TH synthesis in thyrocytes. In this case, a compensatory mechanism was triggered, which included, with the combined use of drugs, an increase in the *Dio3* gene expression and, in the D1 + TRH and D1 + TP + TRH groups, a decrease in the expression of the TSH receptor gene.

### 2.6. Effect of Single-Dose Administration of TPY3m on Hypothalamic and Pituitary Gene Expression in Diabetic Rats

The study of the expression of the pituitary and hypothalamic genes involved in the functioning of the HPT axis did not reveal a significant effect of TPY3m on them. In the D1 + TP group, the expression of the pituitary genes *Tsh-beta* and *Trhr1* encoding the β-subunit of TSH and the TRH receptor; the hypothalamic genes *Pro-TRH*, *Dio2*, and *Dio3* encoding pro-thyroliberin; the TRH precursor; and both forms of deiodinases did not differ significantly from that in the C1 and D1 groups (Table 4). In the D1 + TRH and D1 + TP + TRH groups, the expression of these genes was also not changed, with the exception of an increase in the expression of the β-subunit gene of TSH compared to the diabetic group, which is due to the direct stimulating effect of TRH on the synthesis and secretion of this hormone by pituitary thyrotrophs, as well as an increase in the expression of the *Dio3* gene in the D1 + TRH group (Table 4).

### 2.7. Effect of Three-Day Administration of TPY3m and Levothyroxine on Blood Thyroid Hormone Levels and Thyroid, Pituitary, and Hypothalamic Gene Expression in Diabetic Rats

**Figure 4:**
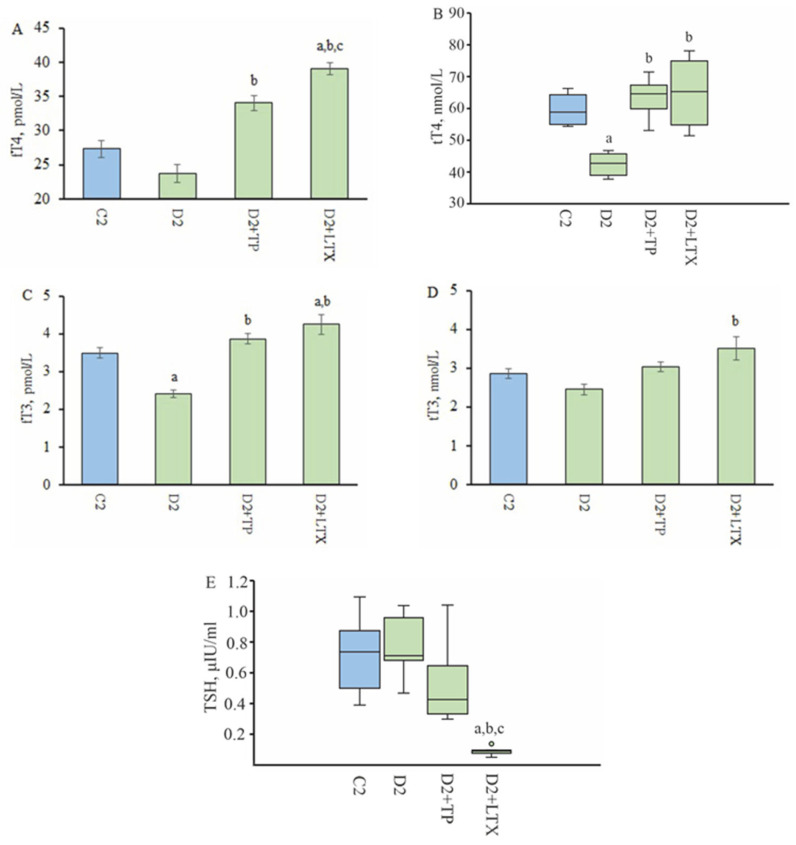
Effect of three-day treatment with TPY3m and levothyroxine on the blood levels of thyroid hormones and TSH in male rats with T2DM. (**A**)—fT4, (**B**)—tT4, (**C**)—fT3, (**D**)—tT3, (**E**)—TSH. Differences with the C2 (^a^), D2 (^b^), and D2 + TP (^c^) groups are significant at *p* < 0.05. The data on the blood tT4 and TSH levels are not normally distributed and are presented as median and interquartile ranges (25%; 75%). The data on the blood levels of fT4, fT3 and tT3 are normally distributed and are presented as M ± SEM. In all groups, *n* = 6.

When administered to diabetic rats for three days, TPY3m significantly increased fT4, tT4, and fT3 levels but had no effect on blood TSH levels (Figure 4). Levothyroxine (orally, 200 μg/kg/day) increased blood TH concentrations, with fT4 and fT3 levels being higher than those in both the D2 and control groups. It suppressed the production of TSH, and the level of this hormone in the blood was eight times lower than in the control and diabetic groups (Figure 4).

In comparison with the group D2, TPY3m, when administered for three days, increased the *Tg*, *Tpo*, and *Dio3* gene expression in the TG, with the *Dio3* expression increasing by an average of four times, and normalized the expression of the TSH receptor gene, which tended to increase in T2DM rats (Table 5). Three-day levothyroxine therapy, although normalizing the thyroid status, suppressed the expression of thyroglobulin and sodium/iodide symporter genes, thereby preventing the production of endogenous THs, and increased the expression of the type 3 deiodinase gene, which degrades THs, by more than five times (Table 5).

After three-day administration, levothyroxine significantly decreased the expression of the *Tsh-beta* and *Trhr1* genes in the pituitary gland, while TPY3m had no significant effect on them (Table 5). In the hypothalamus, TPY3m and levothyroxine treatment decreased the expression of the *Pro-TRH* gene, which was increased in T2DM (Table 5). Thus, TPY3m normalized TRH expression in hypothalamic neurons, which may be a consequence of TPY3m-induced increase in TH levels, which are significantly reduced in T2DM. However, its effect did not extend to the expression of pituitary genes encoding the TRH receptor and TSH, which determine the response of thyrotrophs to TRH stimulation. At the same time, levothyroxine suppressed the expression of these genes, reducing it to 4% in the case of the *Tsh-beta* gene and 35% in the case of the *Trhr1* gene, compared to their expression in the control taken as 100% (Table 5).

### 2.8. Thyroid Tissue Morphology in Diabetic Rats Treated with TPY3m for Three Days

**Figure 5:**
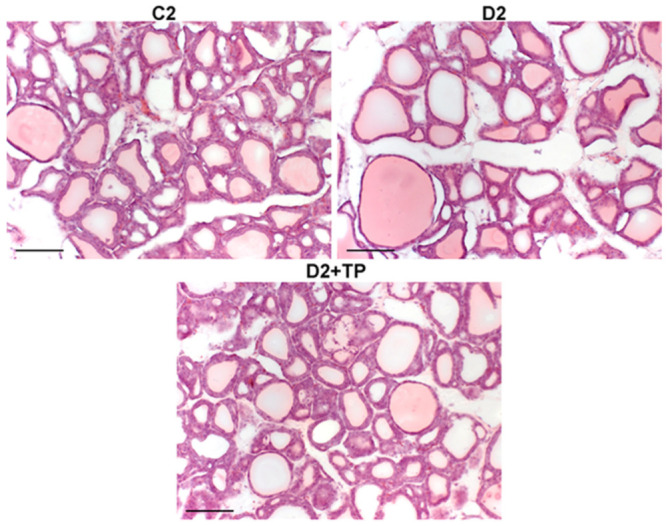
Thyroid gland sections from control (C2) and diabetic rats (D2) and diabetic animals treated with TPY3m for three days (D2 + TP). Hematoxylin and eosin staining. Scale bar: 100 μm. A detailed description of the morphological features is given in the text.

In the control rats, the central and peripheral zones of the TG contained predominantly small oval or round follicles filled with a pale pink colloid bound by cubic or prismatic thyroid epithelium (Figure 5). The thyrocyte nuclei were round or oval and mainly occupied the central area of the cell. The sinusoidal capillaries between the follicles were moderately dilated and contained a small number of formed elements of the blood. A small number of interfollicular islets and small round follicles without a colloid bound by cubic epithelium were detected between the follicles. In the peripheral zone of the TG, single large follicles filled with a dense colloid were found. The thyroid epithelium consists of flat thyrocytes with flat dense nuclei extended along the basement membrane (Figure 5). Thus, the morphological study of the TG in the control rats indicates a moderate level of its functional activity.

In comparison with the control, in rats with T2DM, a greater number of large follicles with flat epithelium and flat nuclei extended along the basement membrane were detected in the peripheral zone of the TG (Figure 5). The development of follicular hypertrophy and accumulation of colloids in thyroid follicles are supported by the data of morphometric analysis, showing a significant increase in the colloid area in the peripheral (but not in the central) zone of the TG (Table 6). The main part of the TG was represented by small groups of oval and round follicles of various sizes, separated by layers of connective tissue. The epithelium in the follicles was mainly flat, less often cubic, and small interfollicular islets were detected between the follicles. Sinusoidal capillaries with formed elements of blood were moderately dilated and detected unevenly (Figure 5). The height of the thyroid follicular epithelium in diabetic rats in both the central and peripheral zones was significantly less than in control animals, which indicates a decrease in the functional activity of the thyroid follicles (Table 6). These data indicate a decrease in the TG activity in rats with T2DM vs. the control.

Rats with T2DM treated with TPY3m for three days showed a more homogeneous thyroid structure compared to untreated diabetic rats. In the peripheral zone of the TG, individual large follicles with a dense bright pink colloid surrounded by flat epithelium were detected (Figure 5). The central zone was represented by small round and oval follicles filled with a pale pink colloid. Follicles with cuboidal epithelium and round nuclei located in the center or basal part of thyrocytes were detected. Interfollicular islets were seen between the follicles, and the sinusoidal capillaries were moderately dilated, containing the formed elements of blood (Figure 5). When treated with TPY3m, in comparison with the D2 group, an increase in the height of the thyroid follicular epithelium and a decrease in the colloid area were shown in the central and peripheral zones of the TG (Table 6). The obtained data indicate that three-day treatment with TPY3m increases the functional activity of the TG in T2DM.

## 3. Discussion

Patients with T2DM are characterized by a wide range of thyroid pathologies, with subclinical and primary hypothyroidism predominating in this case [33,34]. Although some diabetic patients have reduced TH levels, which is usually accompanied by an increase in the TSH level, most of them have TH levels in the reference range [35,36]. In rat models of T2DM, due to their differences from human T2DM both in pathogenesis and the mechanisms of the development of complications of the thyroid system, a characteristic feature of T2DM-induced hypothyroidism is a deficiency of THs [37,38].

Chinese scientists showed that rats with a T2DM induced by a 5-week high-fat diet and an injection of a medium-dose STZ (35 mg/kg) had a significant decrease in fT4 levels, an increase in TSH level and no significant changes in fT3 levels in the blood [37]. Nigerian scientists studied the short-term T2DM induced by a two-week high-fat diet and STZ (35 mg/kg) and demonstrated a strong decrease in T3 levels without a significant change in T4 levels, which was accompanied by a decrease in blood TSH levels [38]. In models of STZ- and alloxan-induced DM, which are more similar to human type 1 DM, a decrease in the levels of all forms of THs was shown [39,40,41,42,43,44]. When STZ was used at a dose of 45 mg/kg during standard diet consumption, a T3 deficiency was demonstrated, but the T4 levels did not change in this case [45]. At the same time, in a rat model of obesity induced by an 8-week high-fat diet without STZ treatment, an increase in TSH levels was observed without significant changes in TH levels [46]. Zucker rats with high-fat diet-induced obesity also showed no thyroid deficiency [47].

In rats with T2DM, we have shown a significant decrease in tT4 and fT3 levels and a tendency to decrease in fT4 and tT3 levels, which indicates impaired TH synthesis (Figure 3 and Figure 4). At the same time, in diabetic rats, the expression of thyroid genes responsible for TH production did not change, with the exception of a tendency to decrease the expression of the thyroglobulin gene (Table 3 and Table 5). The blood TSH level in diabetic rats was similar to that in the control, indicating the absence of significant changes in the functional activity of the hypothalamic and pituitary links of the HPT axis (Figure 4). This is confirmed by the preserved expression of pituitary genes encoding the β-subunit of TSH and the TRH receptor, as well as a moderate increase in the expression of the pro-thyroliberin gene in the hypothalamus of rats with T2DM (Table 4 and Table 5). The differences between the thyroid status in rats with the T2DM model induced by us and that in rats with T2DM models described by other authors also induced by a high-fat diet and STZ [37,38] may be due to the following factors: First, the duration of the high-fat diet differs significantly, and in our case, it is 16 weeks, while other authors have a diet duration of only 2 to 5 weeks. Secondly, we treated the rats with STZ after 10 weeks of a high-fat diet when the animals had already developed obesity and insulin resistance, while, with a short-term high-fat diet, such changes do not have time to develop. It should be noted that in our case, STZ was administered to rats that had an average body weight of 400–430 g. Thus, despite the low dose of STZ (25 mg/kg), its total amount per rat was higher than when STZ was administered to animals with a lower body weight, as shown in other studies. Thus, the damaging effect of STZ on insulin-producing pancreatic β-cells was enhanced, and this caused a more severe form of T2DM, which is illustrated by more pronounced changes in metabolic and hormonal parameters, including the blood TH levels.

To regulate thyroid function, we developed thieno[2,3-d]-pyrimidine derivatives with selectivity for the TSH receptor, the prototype of which was the compound Org41841. A number of them demonstrated the activity of allosteric inverse agonists and antagonists of the TSH receptor [48,49,50], while the compound TPY3m showed the activity of a partial agonist [31,32]. This was supported by an increase in the expression of genes responsible for the synthesis of THs during the incubation of FRTL-5 rat thyrocytes in a TPY3m-containing medium [31], as well as an increase in the blood TH levels in rats upon intraperitoneal and oral administration of TPY3m [32].

In the present study, additional evidence was obtained for the activity of TPY3m as a partial agonist of the TSH receptor. In vitro, TPY3m dose-dependently increased the activity of AC in thyroid membranes isolated from the TG of healthy rats (Figure 1). As is known, in thyroid membranes, stimulation of the TSH receptor leads to the activation and dissociation of the heterotrimeric G_s_ protein functionally coupled to this receptor, as a result of which the Gα_s_ subunit released from the complex activates AC. This leads to an increase in the intracellular level of cAMP, which mediates many regulatory effects of TSH receptor agonists, including the stimulation of the expression and activity of proteins responsible for TH synthesis. We have shown that in the presence of TPY3m, the stimulating effect of TSH on AC activity is not only preserved, but at TPY3m concentrations lower than the EC_50_ value, it is enhanced (Figure 1), and this allows us to classify TPY3m as a positive allosteric modulator (PAM).

The preservation and even the enhancement of the effect of TSH in the presence of TPY3m may be due to differences in the localization of their binding sites, such as the high-affinity orthosteric site to which TSH binds and the allosteric site located inside the transmembrane channel, a target for thieno[2,3-d]-pyrimidine derivatives. The distinct localization and lack of overlap between the extracellular orthosteric and transmembrane allosteric sites have been previously demonstrated by various methods for both the TSH receptor and its related LH receptor [17,51,52,53]. It should be emphasized that a change in the conformation of the transmembrane allosteric site upon binding to the ligand can both weaken (negative allosteric regulation) and facilitate (positive allosteric regulation) signal transduction from the TSH-bound ectodomain to the cytoplasmic regions of the TSH receptor involved in the interaction with heterotrimeric G proteins and β-arrestins [54,55].

The study of the binding of thieno[2,3-d]-pyrimidine derivatives and a number of other compounds with the TSH receptor and its mutant forms [18,51,52,53,56,57,58,59,60,61,62,63] made it possible to establish the structure of the transmembrane allosteric site of the receptor. It is shown to be localized in the upper part of the transmembrane channel, and its cavity is formed by the inner surfaces of five transmembrane helices (TM3, TM4, TM5, TM6, and TM7) and covered from above by segments of the second extracellular loop [64,65,66]. To bind to this site, ligands must overcome the narrow entrance to the transmembrane channel formed predominantly by hydrophobic amino acids. As a result, these ligands must have a pronounced hydrophobicity, which is typical for the compound we developed (TPY3m) [66]. This distinguishes the regulators of the transmembrane allosteric site of the TSH receptor from that of the LH receptor, where the outer entrance to the transmembrane channel is wider and includes amino acids with lower hydrophobicity. It is important that after the translocation of the ligand to the transmembrane allosteric site of the TSH receptor, its hydrophobic part retains the ability to form contacts with the outer mouth of the transmembrane channel, and this largely predetermines the pharmacological profile of the ligand [66,67,68].

Our study on the stimulatory effect of TPY3m on blood TH levels and thyroid gene expression in rats is consistent with the results of the in vitro experiments showing that the AC-stimulating TSH signal is maintained in the presence of TPY3m. Moreover, despite the absence of significant differences between the TH levels in the C + TRH and C + TP + TRH groups, there was a trend toward an enhanced stimulatory effect of TRH on TH production in TPY3m-pretreated rats (Figure 2). The allosteric agonist, NCGC00165237-01, 3-(furan-2-ylmethyl)-2-(4-methoxy-3-(phenoxymethyl)phenyl)-2,3-dihydroquinazolin-4(1H)-one, developed by Susanne Neumann et al. in 2009, also increased basal TH levels and stimulated *Tg* and *Tpo* gene expression in the TG when administered orally and intraperitoneally to mice, but its effect on TSH-stimulated TH levels has not been studied [52], which does not allow for the evaluation of its pharmacological potential on TSH-stimulated TH production.

The activity of TPY3m as an allosteric agonist of the TSH receptor and possibly as an agonist/positive allosteric modulator (ago-PAM) became the starting point for its use to stimulate TH production and correct thyroid deficiency in T2DM. It should be emphasized that the therapeutic potential of low-molecular-weight TSH receptor regulators with agonist activity in hypothyroidism has not been previously assessed. The compound NCGC00165237-01 was studied only as a stimulator of radioactive iodine uptake by thyrocytes in euthyroid mice for its potential use in the diagnosis and treatment of thyroid cancer with radioiodine [52].

Using TPY3m, we were the first to establish that low-molecular-weight allosteric agonists of the TSH receptor are capable of normalizing TH levels reduced in T2DM. The stimulating effect of TPY3m persisted after three-day administration of the drug, indicating the absence of the development of TSH receptor resistance to this compound with its repeated administration. In diabetic rats, treatment with TPY3m demonstrated a significant increase in the expression of a number of thyroid genes involved in the synthesis and degradation of THs, and the pattern of gene expression after a single and three-day administration of TPY3m had some differences. If a single administration significantly increased the expression of *Tg* and *Nis* genes, then a three-day administration increased the expression of *Tg*, *Tpo*, and *Dio3* genes (Table 3 and Table 5). Increased expression of *Tg*, *Nis*, and *Tpo* genes was consistent with increased T4 production resulting from the TPY3m-mediated stimulation of the TSH receptor and activation of AC signaling pathways in thyrocytes. In this regard, it should be noted that there is evidence that a signaling cascade involving the activated TSH receptor, G_s_ protein, AC, protein kinase A, and the transcription factor CREB mediates positive TSH-mediated regulation of the expression of these genes [69,70,71,72].

In the TG of diabetic rats treated with TPY3m for three days, a significant increase in the gene expression of type 3 deiodinase, which degrades T4 and T3 into inactive forms of THs (reverse T3 and diiodothyronine) was shown (Table 5). This is believed to be due to a TPY3m-induced increase in blood T3 levels, which triggers the stimulation and expression of this gene [73,74]. It should be noted that if hypothyroidism is usually characterized by an increase in the ratio of activity and expression of deiodinases of types 2 and 3 [75,76,77], then with the correction of thyroid deficiency using replacement therapy or activators of TH synthesis, this ratio decreases. In our case, the *Dio2*/*Dio3* expression ratio in T2DM rats was increased by an average of 1.5 times compared to the control, while after their three-day treatment with TPY3m, it decreased more than twofold (Table 5). In the case of treatment of diabetic rats with levothyroxine, an even greater decrease in the *Dio2*/*Dio3* expression ratio (about seven times compared to the control) was shown. Along with this, levothyroxine-treated rats showed a significant decrease in the expression of *Tg* and *Nis* genes. This was associated with a decrease in TSH production due to the levothyroxine-mediated suppression of the activity of the hypothalamic and pituitary links of the HPT axis. In the D2 + LTX group, compared with the control and diabetic groups, the expression of the pituitary genes encoding the β-subunit of TSH and the TRH receptor was significantly reduced, and in the hypothalamus, compared with the diabetic group, the expression of the gene encoding pro-TRH was reduced (Table 5). This indicates that even a relatively short course of levothyroxine is able to reduce the activity of the HPT axis, while this does not occur when treating animals with TPY3m.

As noted above, in control rats, TPY3m retained the stimulating effects of endogenous TSH on TH production. Testing the combined effect of TPY3m and TRH in diabetic rats showed that these effects were retained in this case as well. It should be emphasized that in rats with T2DM, the thyroid system response to TRH stimulation did not differ significantly from that in control animals. Thus, TRH-induced increases in the fT4, tT4, fT3, and tT3 levels in the diabetic group averaged 121, 91, 119, and 94% of those in the control group. Based on this, we can conclude that the low-molecular-weight allosteric agonist of the TSH receptor developed by us does not have an inhibitory effect on TSH-mediated TH production not only in healthy but also in diabetic animals. It can be assumed that excessive protein glycation, endoplasmic reticulum stress, and the activation of oxidative and inflammatory processes characteristic of T2DM do not significantly affect the functional activity of the ligand-occupied extracellular orthosteric site and transmembrane allosteric site and the interaction between them. The absence of a negative effect of low-molecular-weight TSH receptor agonists on endogenous TSH-mediated signaling may be of great importance for the development of approaches using such agonists for the treatment of hypothyroidism in metabolic diseases.

Our morphological and morphometric studies demonstrate that rats with T2DM have structural changes in the TG. We have shown an increase in the size of thyroid follicles and the area of colloids in them, as well as a decrease in the height of the thyroid follicular epithelium (Table 6). According to observations by other authors [78,79,80,81], these changes indicate a weakening of their functional activity and are in good agreement with a decrease in TH production in T2DM. Similar structural changes in the TG of rodents with various models of diabetic pathology, including STZ-induced diabetes, have been described in other studies [40,82]. Three-day administration of TPY3m to diabetic rats resulted in an increase in the functional activity of TG, which is consistent with the stimulating effect of TPY3m on TH production. This is evidenced by the appearance of cubic thyrocytes in the follicles in the central zone of the TG and the normalization of the height of the thyroid follicular epithelium and colloid area in the follicles. It is important that TPY3m treatment did not cause destructive changes in the TG. It should be noted that the morphological data we obtained earlier with a five-day administration of TPY3m to healthy rats also indicate that this agonist activates thyroid function [32]. Meanwhile, no signs of thyroid degradation in the left and right lobes and infiltration of lymphoid elements into them were detected, which indicates the absence of a negative effect of the long-term treatment of animals with TPY3m on the TG structure [32].

## 4. Materials and Methods

### 4.1. Synthesis and Physicochemical Characterization of the Compound TPY3m

The compound TPY3m, ethyl-2-(4-(4-(5-amino-6-(*tert*-butylcarbamoyl)-2-(methylthio)thieno[2,3-d]pyrimidin-4-yl)phenyl)-*1H*-1,2,3-triazol-1-yl) acetate, was synthesized by reacting 5-amino-*N*-(*tert*-butyl)-4-(4-ethynylphenyl)-2-(methylthio)thieno[2,3-d]pyrimidine-6-carboxamide (1) and ethyl 2-azidoacetate (2) in the presence of a copper-containing catalyst (3), as described by us previously [31] (Figure S2). The melting point for the TPY3m was 218.1–218.6 °C.

Physicochemical characterization of the target product was carried out using ^1^H-NMR and ^13^C-NMR (“Bruker Avance III 400”, Hamburg, Germany) and high-resolution mass spectrometry (“LCMS-9030”, Kyoto, Japan).

^1^H-NMR spectrum (frequency 400 MHz, solvent—deuterated chloroform): δ 8.09—8.03 (m, 3H Ar phenyl + Ar triazol), 7.76 (d, *J* = 7.9 Hz, 2H Ar phenyl), 5.32 (s, 2H CArCH2C(O)), 5.26 (m, 3H NH2 + NH), 4.34 (q, *J* = 7.1 Hz, 2H CH2 C(O)OEt), 2.69 (s, 3H MeS), 1.48 (s, 9H tBu), and 1.36 (t, *J* = 7.2 Hz, 3H CH3 C(O)OEt) (Figure S3).

^13^C-NMR spectrum (frequency 101 MHz, solvent—deuterated chloroform): δ 169.74, 167.65, 166.28, 165.14, 162.13, 147.27, 144.74, 135.96, 132.77, 129.85, 126.32, 121.74, 117.86, 97.10, 62.77, 52.28, 51.28, 29.27, 14.63, and 14.26 (Figure S4).

High resolution mass spectrum (ESI+, 100 V, MeOH): found—*m*/*z* 526.1695 [M + H]^+^; calculated for C_24_H_28_N_7_O_3_S_2_^+^—*m*/*z* 526.1690 (Figure S5).

### 4.2. Animals

Male Wistar rats (*Rattus norvegicus*) were obtained from the “Rappolovo” animal nursery (Leningrad Region, Russia). The animals were housed in plastic cages, with six rats in each, with a 12 h light/12 h dark cycle and at a temperature of 23 ± 2 °C. They had free access to laboratory chow pellets and drinking water. The daily standard diet contained 20–25 g of dry food. Before the start of the experiments, all animals were acclimatized for one week, for which they were placed in cages with a capacity of up to 6 animals each and fed standard dry food.

The experimental procedures were approved by the Institutional Animal Care and Use Committee at the Sechenov Institute of Evolutionary Physiology and Biochemistry (St. Petersburg, Russia) (protocol number 4-2/2024, session 4 of 25 April 2024) and according to “The Guide for the Care and Use of Laboratory Animals” and the European Communities Council Directive recommendations for the care and use of laboratory animals (2010/63/EU). All efforts to minimize animal suffering and reduce the number of rats used in experiments were made. When collecting blood from the tail vein for the evaluation of biochemical parameters, local anesthesia was used with a 2% Lidocaine solution (2–4 mg/kg b.w.). When decapitating animals, general anesthesia was used, which was induced by the intraperitoneal administration of chloral hydrate (400 mg/kg b.w.) to the rats.

### 4.3. Isolation of Rat Thyroid and Testicular Membranes

To obtain thyroid membrane fractions, adult male Wistar rats without any signs of pathology were taken. Animals were decapitated under anesthesia (chloral hydrate, 400 mg/kg b.w., i.p.), after which the thyroid tissues were quickly removed for the further isolation of thyroid membranes. Plasma membrane fractions from the rat TGs were obtained according to the method described previously [83,84], with some of our modifications [85]. Isolated thyroid tissue was cut into small pieces and washed in ice-cold 40 mM of Tris-HCl buffer (pH 7.4) containing 4 mM of MgCl_2_ (Buffer A). The TG pieces were homogenized using a Polytron in 10 volumes of Buffer A containing the protease inhibitors (2 μM of pepstatin, 1 mM of leupeptin, 20 μg/mL of aprotinin, and 100 μM of phenylmethylsulfonyl fluoride). The resulting homogenate was filtered through a triple layer of gauze, after which the filtrate was thoroughly homogenized in a glass homogenizer with cooling in an ice bath and then centrifuged for 15 min at 800× *g* at 4 °C. The pellets were discarded, and the supernatant was centrifuged at 26,500× *g* for 30 min at 4 °C. The final pellets were resuspended in Buffer A to produce the membrane fraction with a protein concentration range of 1–2 mg/mL and stored at −80 °C. The protein concentration of each membrane preparation was measured according to the method of Lowry and colleagues using BSA as a standard [86].

To obtain testicular membrane fractions, the rats were decapitated under anesthesia (chloral hydrate, 400 mg/kg b.w., i.p.), and their testes were removed. The testes tissue was crushed and placed in ice-cold Buffer A containing 320 mM of sucrose and a cocktail of protease inhibitors. The tissue samples were homogenized with a Polytron, and the homogenate was centrifuged at 1500× *g* for 10 min at 4 °C. The supernatant was centrifuged at 20,000× *g* for 30 min at 4 °C. The resulting pellets were washed by resuspension in 10 volumes of Buffer A without sucrose and centrifuged at 20,000× *g* for 30 min at 4 °C. The final pellets obtained from the rat testes were resuspended and stored in the same manner as for the TG pellets.

### 4.4. Determination of Basal and TSH Receptor Agonist-Stimulated Adenylate Cyclase Activity in Thyroid Membranes

The activity of adenylate cyclase (AC) (ATP diphosphate-lyase, 3′,5′-cyclic-AMP-forming; EC 4.6.1.1) in thyroidal membranes was measured according to the method of Yoram Salomon and colleagues [87], with some modifications [88]. The incubation mixture for measuring AC activity contained 50 mM of Tris-HCl (pH 7.5), 5 mM of MgCl_2_, 1 mM of ATP, 0.1 mM of cAMP, 20 mM of creatine phosphate, 0.2 mg/mL of creatine phosphokinase, a radioactively labeled substrate—1.0 μCi [α-^32^P]-ATP, and 50–100 μg of the membrane protein (final volume of 100 μL). All reagents for the enzymatic reaction were obtained from “Sigma-Aldrich” (St. Louis, MO, USA), while [α-^32^P]-ATP was obtained from “Izotope” (Moscow, Russia). The reaction was carried out at 37 °C for 12 min. To stop the reaction, 100 μL of 0.5 M of HCl was used, after which the tubes were placed in a boiling water bath for 6 min. After boiling, 100 μL of 1.5 M of imidazole was added to each tube to neutralize the hydrochloric acid. The [^32^P]-cAMP formed during the reaction was separated from other labeled products by column chromatography. For this, the samples were placed on columns with neutral alumina, and the labeled cAMP was eluted with 10 mM of imidazole–HCl buffer (pH 7.4). The eluates were collected in scintillation vials and counted using a 1209/1215 RackBeta scintillation counter (“LKB”, Stockholm, Sweden). Each assay was carried out in triplicate at least three times, and the results are presented as pmol cAMP/min per mg of membrane protein. The basal AC activity in the thyroidal membranes was measured in the absence of TSH or TPY3m. TPY3m was added to the incubation mixture as a solution in 2.5 μL of DMSO, which constituted 2.5% DMSO of the total volume of the incubation mixture and did not have a significant effect on AC activity. The same volume of DMSO was added to the control samples and samples with the addition of TSH alone. When studying the combined action of TPY3m and TSH, the thyroid membranes were first incubated with TPY3m for 5 min at 4 °C to distribute this compound into the membrane fraction, after which TSH was added, and the incubation mixture was placed in a water bath and incubated at 37 °C for 12 min to carry out the enzymatic reaction. The EC_50_ value for the AC-stimulating effects of TPY3m was calculated using the algorithm presented at https://www.aatbio.com/tools/ec50-calculator, accessed on 17 September 2024. 

### 4.5. Determination of the Level of Hormones, Glucose, Glycated Hemoglobin, and Lipids in the Blood of Rats

The blood levels of TSH, insulin, and leptin were measured with a “Rat Thyroid Stimulating Hormone ELISA kit” (“Cusabio Biotech Co., Ltd.”, Wuhan, China), “Rat Insulin ELISA kit” (“Mercodia AB”, Uppsala, Sweden), and “ELISA for Leptin, Rat” (“Cloud-Clone Corp.”, Houston, TX, USA), respectively. The blood levels of free thyroxin (fT4), total thyroxine (tT4), free triiodothyronine (fT3), and total triiodothyronine (tT3) were determined by ELISA kits obtained from “Immunotech” (Moscow, Russia). The level of testosterone in the blood was assessed using a “Testosterone-ELISA kit” from “AlkorBio” (Saint-Petersburg, Russia). The blood glucose levels were measured using a glucometer (“Life Scan Johnson & Johnson”, Milpitas, CA, USA) and the test-strips “One Touch Ultra” (“LifeScan Inc.”, Malvern, PA, USA). The content of glycated hemoglobin (HbA1c) was assessed using a “Multi Test HbA1c System kit” (“Polymer Technology Systems, Inc.”, Indianapolis, IN, USA). The blood levels of triglycerides and total cholesterol were assessed using the test strips “MulticareIN” from “Biochemical Systems International S.p.A.” (Arezzo, Italy).

### 4.6. Intraperitoneal Glucose Tolerance Test

To assess impaired glucose tolerance as an important marker for the development of T2DM, an intraperitoneal glucose tolerance test (IGTT) was performed. For this, rats were given a single intraperitoneal injection of glucose at a dose of 2 g/kg of body weight, as described previously [89]. Before the test, the animals were fasted for 10 h (the cages were cleared of possible food residues) with free access to water. Blood glucose levels were measured for 120 min: before the glucose load and 15, 30, 60, 90, and 120 min after it. Based on the obtained data, the AUC_0–120_ values were calculated for the curve “glucose concentration, mM—time, minutes”, an increase which, compared with those in the control, indicated the degree of impaired glucose tolerance. Insulin and leptin levels were assessed before and 120 min after the glucose load. Blood was collected from the tail vein using local anesthesia (2% Lidocaine solution, 2–4 mg/kg b.w.). The IGTT was performed at least a week before experiments with the TSH receptor regulators to avoid a possible effect of the glucose load on the HPT axis parameters.

### 4.7. Induction of Type 2 Diabetes Mellitus in Male Rats

A rat model of T2DM was induced using a combination of a saturated fat-rich diet and a single STZ treatment, as described previously [89]. The high-fat diet included daily consumption by rats of 5–7 g of a fat mixture containing (*w*/*w*) 52.4% pork lard, 41.7% curd, 5% liver, 0.5% L-methionine, 0.2% baker’s yeast, and 0.2% NaCl [89]. The animals had free access to dry pelleted food (standard diet), which was consumed by the control rats. Daily consumption of dry pelleted food (19% protein, 5% fat, 4% fiber, and 9% ash) was 20–25 g, which provided 2.95 kcal/g. To develop T2DM, two-month-old male rats were placed on a high-fat diet for 16 weeks, and after the 10th week of the diet, the animals were treated with STZ (“Sigma-Aldrich”, St. Louis, MO, USA). STZ was administered at a dose of 25 mg/kg (intraperitoneally in 0.1 M of sodium–citrate buffer at pH 4.5) to decompensate the insulin-producing function of the pancreas. After the STZ injection, the body weight and postprandial blood glucose levels were monitored weekly. Five weeks after the STZ injection (one week before the end of the high-fat diet), the animals were tested for T2DM markers (an assessment of glucose levels before and 120 min after the glucose load, as well as insulin and leptin levels before and 120 min after the glucose load in the IGTT). Body weight gain, glycated hemoglobin content, and lipid levels (triglycerides and total cholesterol) were also assessed. Animals with increased body weight (by an average of 10% compared to the control), increased glycated hemoglobin levels (more than 10%), and impaired glucose tolerance in the IGTT were considered diabetic, after which they were randomly assigned to groups and used in further experiments. Overall, T2DM developed in at least 65% of animals that were fed a high-fat diet and received a low-dose STZ injection.

### 4.8. Treatment of Rats with TPY3m, Thyroliberin, and Levothyroxine

TPY3m was administered to rats intraperitoneally at a dose of 20 mg/kg of body weight, both as a single and three-day administration. The drug was dissolved in DMSO (200 μL). The preliminary studies showed that the volume of DMSO used did not have a toxic effect on the animals or affect the TH levels and the functional state of the HPT axis. Levothyroxine was given orally (using a gastric tube) at a dose of 200 μg/kg/day for three days. Treatment with both drugs was carried out at 10:00 am. TRH (“Sigma-Aldrich”, St. Louis, MO, USA), a TSH releasing factor, was administered intranasally once at a dose of 100 μg/rat 30 min after TPY3m administration. For this purpose, TRH was dissolved in 20 μL of physiological solution and administered dropwise into both nostrils (10 μL into each nostril). The animals were in the spinal position. Control animals received solvents instead of the studied drugs.

### 4.9. Animal Groups and Their Treatment

Series 1: To evaluate the effect of TPY3m on the basal and TRH-stimulated thyroid system, healthy male rats were divided into 4 groups (6 animals in each): control rats (C), which received solvents instead of drugs; group C + TRH, which received TRH at 10:30 a.m. (intranasally, 100 μg/rat); group C + TP, which received TPY3m at 10:00 a.m. (intraperitoneally, 20 mg/kg); and group C + TP + TRH, which received TPY3m and TRH sequentially at the same times and doses.

Series 2: To study the effect of TPY3m on the basal and TRH-stimulated thyroid system in male rats with T2DM, 5 groups (6 animals in each) were formed: control (C1) and diabetic (D1) rats, which received solvents instead of drugs; group D1 + TRH, diabetic rats, which received TRH at 10.30 a.m. (intranasally, 100 μg/rat); group D1 + TP, diabetic rats, which received TPY3m at 10.00 a.m. (intraperitoneally, 20 mg/kg); and group D1 + TP + TRH, diabetic rats, which received TPY3m and TRH sequentially at the same times and doses.

Series 3: For a comparative study of the effect of TPY3m and levothyroxine on the thyroid system in male rats with T2DM, 4 groups were formed (6 animals in each): control (C2) and diabetic (D2) rats, which received solvents instead of drugs for 3 days; group D2 + TP, diabetic rats, which received TPY3m daily at 10:00 a.m. (intraperitoneally, 20 mg/kg/day) for 3 days; and group D2 + LTX, diabetic rats, which received levothyroxine daily at 10:00 a.m. (orally through a gastric tube, 200 μg/kg/day) for 3 days. Throughout all three days of the experiment, animals in the diabetic groups continued to receive a high-fat diet.

At the end of the experiment (3.5 h after the last administration of TPY3m or its vehicle), the animals were anesthetized using chloral hydrate (400 mg/kg b.w., i.p.), after which they were decapitated and tissue samples were collected for RT-PCR (TG, pituitary, and hypothalamus) and morphological analysis (TG).

### 4.10. The Analysis of Gene Expression Using RT-PCR

The total RNA was isolated from the rat tissues (TG, pituitary, and hypothalamus) using the “ExtractRNA Reagent” (“Evrogen”, Moscow, Russia) according to the manufacturer’s instructions, as described earlier [90]. The tissue samples containing 1 μg of RNA were transcribed to cDNA with the random oligodeoxynucleotide primers and the “MMLV-RT Kit” (“Evrogen”, Moscow, Russia). The amplification procedure was carried out using the incubation mixture containing 10 ng of reverse-transcribed product, 0.4 μM of the forward and reverse primers, and a “qPCRmix-HS SYBR + LowROX Kit” (“Evrogen”, Moscow, Russia). Signals were detected using the “Applied Biosystems^®^ 7500 Real-Time PCR System” (“Life Technologies, Thermo Fisher Scientific Inc.”, Waltham, MA, USA). The primers that were used to study the gene expression are presented in Table S2. The obtained data were calculated using the delta–delta C_t_ method [91] and are expressed as fold expression relative to expression in the control group (RQ). The expression of the genes encoding actin B (Actb) and 18S rRNA was used as an endogenous control.

### 4.11. Morphological and Morphometric Analysis of Thyroid Tissue

Thyroid tissues were fixed for 72 h (4 °C) in 15% *para*-formaldehyde in a phosphate buffer (PBS, pH 7.4). After washing in the PBS and cryoprotection in PBS containing 30% sucrose, the thyroid tissue samples were embedded in a Tissue-Tek medium (“Sacura, Finetek Europe”, Alphen aan den Rijn, The Netherlands) and frozen on dry ice. Thyroid sections of 7 μm thickness were prepared using a Leica-1520 cryostat (“Leica Microsystems”, Wetzlar, Germany). Every fifth section was mounted on Super-Frost glass (“Menzel”, Berlin, Germany) coated with gelatin, with sections from the TG of different animal groups mounted on one glass. The glass was dried at room temperature overnight, stained with hematoxylin and eosin, washed in water, and mounted under a cover glass (“Menzel”, Berlin, Germany) using glycerol. Micrographs were obtained using a transmitted light Carl Zeiss Imager A1 (Axio Vision 4.7.2) microscope at ×20 magnification (“Carl Zeiss”, Jena, Germany) and using the Zen 3.4 (blue edition) program.

Morphometric studies were performed on longitudinal sections of the TG with the largest area, according to previously developed approaches [80,92,93]. For quantitative analysis, in each sample, five follicles were collected from each of five different regions of the peripheral or central thyroid zones. In the analyzed follicle, the height of the follicular epithelium (in μm) was determined as the average after measuring four cells located at opposite poles. Along with this, the colloid area (in μm^2^) in the same areas of the peripheral and central zones was estimated. The height of the thyroid follicular epithelium (μm) and the colloid area (μm^2^) were measured using Image J NIH Analysis software, Available online: https://imagej.net; accessed on 24 July 2024 (National Institutes of Health, Bethesda, MD, USA) at a magnification of 200×.

### 4.12. Statistical Analysis

Statistical analysis was performed using IBM SPSS Statistics 26 (“IBM”, Armonk, NY, USA). The normality of distribution was tested via the Shapiro–Wilk test, while Livigne’s test was used for the equality of variances. Some of the data was not normally distributed (the in vivo effects of TPY3m and thyroliberin on the plasma levels of some THs and expression of some thyroid and pituitary genes and the morphometric parameters of the TG). In this case, the comparisons between groups were made using the Kruskal–Wallis H test, followed by an analysis made by pairwise comparisons using the Mann–Whitney U-test, and the data are presented as median and interquartile ranges (25%; 75%). Under normal distribution conditions, the in vitro effects of TSH receptor agonists and the in vivo effects of TPY3m and TRH were performed using one-way ANOVA. Post hoc analysis was performed using Tukey’s test. The data are presented as the mean ± standard error of the mean (M ± SEM). All differences are considered as significant at *p* < 0.05.

## 5. Conclusions

The low-molecular-weight allosteric agonist TPY3m, a thieno[2,3-d]-pyrimidine derivative developed by us, is able to increase the TH level when administered to both healthy male rats and animals with high-fat diet/STZ-induced T2DM. When administered to diabetic animals both once and for three days, TPY3m normalized the levels of T4 and T3 reduced in T2DM rats and did not significantly affect the blood TSH level and the expression of hypothalamic and pituitary genes responsible for the synthesis and secretion of TSH. Thus, TPY3m can be used to compensate for thyroid deficiency in metabolic disorders without a significant inhibitory effect on TSH production.

Pretreatment with TPY3m did not reduce the stimulating effect of TRH on TH production and expression of thyroid genes involved in the synthesis of these hormones in both control and diabetic rats. This indicates that TPY3m does not have an inhibitory effect on the stimulation of TH production by endogenous TSH. It is important that the in vitro experiments demonstrated varying additivity of the stimulating effects of TPY3m and TSH on AC activity in thyroid membranes, and at concentrations of TPY3m below the EC_50_, potentiation of these effects was observed. This may indicate the activity of TPY3m as an ago-PAM and is in good agreement with the data of the in vivo experiments on the preservation and even slight enhancement of TRH-stimulated TH production.

Morphological analysis of the TG in diabetic rats after three-day administration of TPY3m showed an increase in the TG functional activity and demonstrated the absence of destructive changes in TG. This, together with previously obtained data on five-day administration of the drug to healthy animals [32], confirms the activity of TPY3m as a stimulator of thyroid function and indicates the absence of its damaging effect on the TG. The absence of a significant effect of TPY3m in vitro and in vivo on the LH receptor, which is structurally related to the TSH receptor, argues in favor of the receptor specificity of this compound.

In summary, a compound with the activity of an allosteric partial agonist of the TSH receptor has been created, which can become a prototype for pharmacological drugs for the correction of hypothyroidism, including that induced by T2DM and other metabolic and endocrine disorders.

## Figures and Tables

**Table 1:** Effect of TPY3m treatment (single 20 mg/kg, i.p.) on basal and thyroliberin-stimulated expression of genes responsible for thyroid hormone synthesis and TSH receptor gene expression in the thyroid gland of healthy rats.

Gene	C	C + TRH	C + TP	C + TP + TRH
*Tg*	1.03 ± 0.10	1.86 ± 0.20 ^a^	1.64 ± 0.08	2.19 ± 0.28 ^a^
*Tpo*	1.06 (0.75; 1.21)	2.67 (2.20; 4.12) ^a^	1.69 (1.57; 1.98) ^a,b^	2.75 (2.18; 3.67) ^a^
*Nis*	0.99 (0.83; 1.22)	2.09 (1.86; 2.59) ^a^	1.36 (1.17; 1.73) ^b^	2.91 (1.83; 3.91) ^a^
*Dio2*	1.01 ± 0.12	2.21 ± 0.24 ^a^	1.96 ± 0.22 ^a^	2.77 ± 0.32 ^a^
*Tshr*	1.02 ± 0.13	0.78 ± 0.11	0.89 ± 0.09	0.80 ± 0.07

Note: The differences with the control (^a^) and C + TRH (^b^) groups are significant at *p* < 0.05. The data on the *Tpo* and *Nis* gene expression (RQ values) are not normally distributed and are presented as median and interquartile ranges (25%; 75%). The data on the *Tg*, *Dio2*, and *Tshr* gene expression (RQ values) are normally distributed and are presented as M ± SEM. In all groups, *n* = 6.

**Table 2:** Metabolic and hormonal parameters in male rats with type 2 diabetes induced by high-fat diet and low dose of streptozotocin.

Indicator	Control	Diabetes
Body weight, g	417.3 ± 7.9	462.3 ± 6.9 ^a^
Glucose (0, fasting), mM *	4.87 ± 0.26	5.75 ± 0.12 ^a^
Glucose (120), mM **	5.25 ± 0.27	8.13 ± 0.28 ^a^
HbA1c, %	4.17 ± 0.11	6.45 ± 0.18 ^a^
Insulin (0, fasting), ng/mL *	0.83 ± 0.14	1.23 ± 0.11
Insulin (120), ng/mL **	0.92 ± 0.06	2.05 ± 0.12 ^a,b^
IR index (basal), arb. units	4.04 ± 0.73	7.13 ± 0.67 ^a^
IR index (120), arb. units	4.85 ± 0.50	16.38 ± 0.94 ^a,b^
Leptin (0, fasting), ng/mL *	1.38 ± 0.16	2.79 ± 0.23 ^a^
Leptin (120), ng/mL **	1.96 ± 0.34	5.46 ± 0.38 ^a,b^
Triglycerides, mM	1.87 ± 0.13	2.49 ± 0.08 ^a^
Total cholesterol, mM	4.25 ± 0.19	5.33 ± 0.16 ^a^

Notes: The data are presented for diabetic rats, which were then randomly divided into groups D1, D1 + TRH, D1 + TP, and D1 + TP + TRH to evaluate the efficacy of one-day TPY3m treatment in the second series of experiments. * Fasting glucose, insulin, and leptin levels (after 10 h fasting before IGTT) and ** glucose, insulin, and leptin levels 120 min after the glucose load in IGTT. The insulin resistance (IR) indices were calculated both for the baseline state (the product of fasting glucose concentration and insulin concentration before IGTT) and 120 min after the glucose load in IGTT. ^a^ The differences with the control are significant at *p* < 0.05. ^b^ The differences between hormone levels or the IR indices before and after the glucose load in the same group are significant at *p* < 0.05. In the control group, *n* = 6, and in the diabetic group, *n* = 24.

**Table 3:** Effect of single-dose TPY3m administration (20 mg/kg, i.p.) on basal and thyroliberin-stimulated expression of thyroid genes encoding the components of thyroid hormone synthesis system and the TSH receptor in diabetic rats.

Gene	C1	D1	D1 + TRH	D1 + TP	D1 + TP + TRH
*Tg*	1.06 (0.41; 1,58)	0.52 (0.30; 0.67)	1.56 (1.09; 2.05) ^b^	1.42 (1.28; 1.54) ^b^	1.62 (0.81; 1.73) ^b^
*Tpo*	1.04 ± 0.12	0.81 ± 0.11	1.65 ± 0.15 ^b^	1.13 ± 0.14	1.51 ± 0.26 ^a,b^
*Nis*	1.03 ± 0.18	0.98 ± 0.23	1.71 ± 0.27	2.03 ± 0.29 ^b^	2.88 ± 0.24 ^a,b,c^
*Dio2*	0.90 (0.81; 1.38)	1.23 (0.95; 1.49)	1.81 (1.54; 2.21) ^a,b^	1.72 (1.37; 2.06) ^a^	1.99 (1.44; 2.75) ^a^
*Dio3*	1.02 ± 0.20	0.79 ± 0.13	1.98 ± 0.39	2.15 ± 0.34	2.41 ± 0.52 ^b^
*Tshr*	0.92 (0.72; 1.52)	1.23 (0.85; 2.88)	0.49 (0.25; 0.72) ^a,b^	0.90 (0.70; 1.22)	0.37 (0.28; 0.90) ^b^

Note: The differences with the C1 (^a^), D1 (^b^) and D1 + TRH (^c^) groups are significant at *p* < 0.05. The data on the expression (RQ values) of the *Tg*, *Dio2* and *Tshr* genes are not normally distributed and are presented as median and interquartile ranges (25%; 75%). The data (RQ values) on the expression of *Tpo*, *Nis,* and *Dio3* genes are normally distributed and are presented as M ± SEM. In all groups, *n* = 6.

**Table 4:** Effect of single-dose TPY3m administration (20 mg/kg, i.p.) on hypothalamic and pituitary gene expression in diabetic rats.

Gene	C1	D1	D1 + TRH	D1 + TP	D1 + TP + TRH
Pituitary					
*Tsh-beta*	1.08 ± 0.19	0.75 ± 0.09	1.28 ± 0.14 ^b^	1.05 ± 0.07	1.23 ± 0.13 ^b^
*Trhr1*	1.06 ± 0.14	0.95 ± 0.19	0.83 ± 0.19	1.83 ± 0.42	1.12 ± 0.28
Hypothalamus					
*Pro-TRH*	1.04 ± 0.15	1.62 ± 0.27	1.46 ± 0.27	1.55 ± 0.28	1.16 ± 0.31
*Dio2*	1.01 ± 0.16	1.44 ± 0.25	1.85 ± 0.45	1.71 ± 0.18	1.67 ± 0.44
*Dio3*	1.04 ± 0.14	0.83 ± 0.18	2.24 ± 0.44 ^b^	1.81 ± 0.39	1.72 ± 0.33

Note: Differences with the D1 (^b^) group are significant at *p* < 0.05. The data (RQ values) on the expression of hypothalamic and pituitary gene are normally distributed and are presented as M ± SEM, and in all groups, *n* = 6.

**Table 5:** Effect of three-day treatment with TPY3m and levothyroxine on the expression of thyroid, pituitary, and hypothalamic genes in male rats with T2DM.

Gene	C2	D2	D2 + TP	D2 + LTX
Thyroid gland				
*Tg*	1.01 ± 0.19	0.51 ± 0.09	1.46 ± 0.15 ^b^	0.38 ± 0.10 ^a,c^
*Tpo*	1.03 ± 0.12	1.00 ± 0.13	1.55 ± 0.09 ^a,b^	1.03 ± 0.14 ^c^
*Nis*	1.02 ± 0.18	1.16 ± 0.20	1.49 ± 0.18	0.32 ± 0.11 ^a,b,c^
*Dio2*	0.99 ± 0.15	1.32 ± 0.27	1.61 ± 0.31	0.71 ± 0.22
*Dio3*	1.05 (0.88; 1.24)	0.92 (0.48; 1.06)	4.05 (2.17; 4.31) ^a,b^	5.48 (4.11; 7.25) ^a,b^
*Tshr*	1.05 (0.69; 1.31)	1.61 (0.89; 2.48)	0.76 (0.66; 1.01) ^b^	1.44 (0.97; 1.71)
Pituitary				
*Tsh-beta*	1.15 (0.64; 1.21)	0.79 (0.49; 1.11)	0.60 (0.43; 1.12)	0.04 (0.03; 0.05) ^a,b,c^
*Trhr1*	1.05 ± 0.15	0.95 ± 0.16	0.85 ± 0.12	0.36 ± 0.07 ^a,b^
Hypothalamus				
*Pro-TRH*	1.00 ± 0.15	1.80 ± 0.23 ^a^	0.75 ± 0.12 ^b^	0.54 ± 0.10 ^b^
*Dio2*	1.05 ± 0.14	1.51 ± 0.18	1.11 ± 0.14	1.20 ± 0.16
*Dio3*	1.00 ± 0.18	0.67 ± 0.11	0.93 ± 0.12	0.80 ± 0.13

Note: Differences with the C2 (^a^), D2 (^b^), and D2 + TP (^c^) groups are significant at *p* < 0.05. The data on the expression (RQ) of the *Dio3* (thyroid), *Tshr*, and *Tsh-beta* genes are not normally distributed and are presented as median and interquartile ranges (25%; 75%). The data (RQ) on the expression of other genes are normally distributed and are presented as M ± SEM. In all groups, *n* = 6.

**Table 6:** The height of the thyroid follicular epithelium (μm) and the colloid area (μm^2^) in the peripheral and central thyroid zones of control, untreated diabetic, and TPY3m-treated diabetic rats.

Parameter	C2	D2	D2 + TP
	Central zone		
The height of thyroid follicular epithelium (μm)	10.8 (9.7; 11.7)	7.7 (6.6; 8.3) ^a^	11.5 (9.6; 12.7) ^b^
Colloid area (μm^2^)	2658 (1847; 3685)	2883 (1966; 5281)	1270 (887; 1923) ^a,b^
	Peripheral zone		
The height of thyroid follicular epithelium (μm)	9.2 (7.1; 10.2)	5.8 (5.2; 6.6) ^a^	9.6 (8.2; 10.6) ^b^
Colloid area (μm^2^)	5703 (3992; 9489)	12,372 (8693; 14,551) ^a^	3001 (964; 7825) ^b^

Note: Differences with the C2 (^a^) and D2 (^b^) groups are significant at *p* < 0.05. The data are not normally distributed and are presented as median and interquartile ranges (25%; 75%). In all groups, *n* = 6.

## Data Availability

Data are contained within the article.

## Supplementary Materials

The following supporting information can be downloaded at: https://www.mdpi.com/article/10.3390/ijms26020703/s1.

## Abbreviations

AC	adenylate cyclase
fT3	free triiodothyronine
fT4	free thyroxine
G_s_ protein	heterotrimeric G protein of the stimulatory type
HbA1c	glycated hemoglobin
HPT axis	hypothalamic–pituitary–thyroid axis
IGTT	intraperitoneal glucose tolerance test
IR	insulin resistance
LH	luteinizing hormone
PAM	positive allosteric modulator
T2DM	type 2 diabetes mellitus
T3	triiodothyronine
T4	thyroxine
fT3	free triiodothyronine
fT4	free thyroxine
tT3	total triiodothyronine
tT4	total thyroxine
TG	thyroid gland
TH	thyroid hormone
TPY3m	ethyl-2-(4-(4-(5-amino-6-(tert-butylcarbamoyl)-2-(methylthio)thieno[2,3-d]pyrimidin-4-yl)phenyl)-1H-1,2,3-triazol-1-yl) acetate
TRH	thyroid-stimulating hormone-releasing factor
TSH	thyroid-stimulating hormone
STZ	streptozotocin
