# Supplementary material for: Effect of a Low-Molecular-Weight Allosteric Agonist of the Thyroid-Stimulating Hormone Receptor on Basal and Thyroliberin-Stimulated Activity of Thyroid System in Diabetic Rats
Source: Int J Mol Sci. 2025 Jan 15;26(2):703. doi: 10.3390/ijms26020703 (PMC11766125; doi:10.3390/ijms26020703)
Supplement: Supplementary file 1 [file ijms-26-00703-s001.zip › Figure S1 new.pdf]

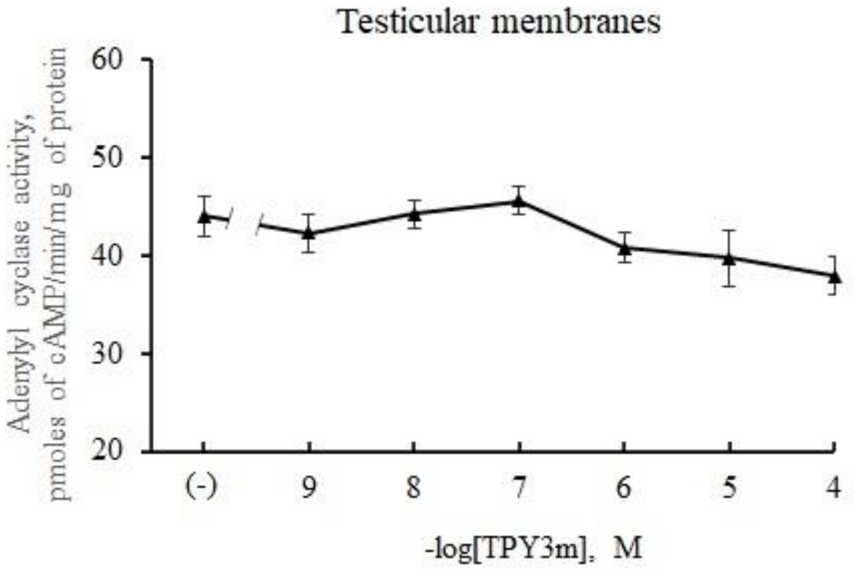

Figure S1. Adenylate cyclase activity in testicular membranes of male rats treated with TPY3m. The basal AC activity in testicular membranes was 44.0±2.1 pmol cAMP/min per mg of membrane protein. The effect of TPY3m on the basal AC activity in testicular membranes was assessed in the concentration range from 10^{-9} to 10^{-4} M.
