# Supplementary material for: Effect of a Low-Molecular-Weight Allosteric Agonist of the Thyroid-Stimulating Hormone Receptor on Basal and Thyroliberin-Stimulated Activity of Thyroid System in Diabetic Rats
Source: Int J Mol Sci. 2025 Jan 15;26(2):703. doi: 10.3390/ijms26020703 (PMC11766125; doi:10.3390/ijms26020703)
Supplement: Supplementary file 1 [file ijms-26-00703-s001.zip › Figure S2.pdf]

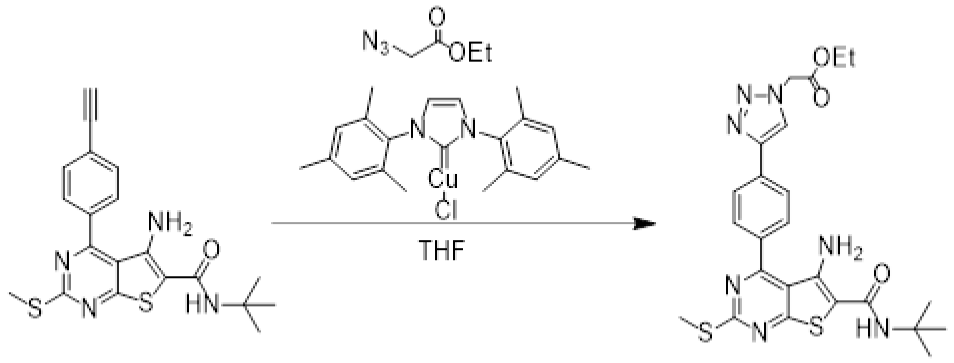

Figure S2. Synthesis of compound TPY3m, ethyl 2-(4-(4-(5-amino-6-(*tert*-butylcarbamoyl)-2-(methylthio)thieno[2,3-d]-pyrimidin-4-yl)phenyl)-*1H*-1,2,3-triazol-1-yl) acetate. The synthesis was carried out by reacting 5-amino-*N*-(*tert*-butyl)-4-(4-ethynylphenyl)-2-(methylthio)thieno[2,3-d]pyrimidine-6-carboxamide (**1**) and ethyl 2-azidoacetate (**2**) in tetrahydrofuran (THF) in the presence of a copper-containing catalyst (**3**).
