# Supplementary material for: Effect of a Low-Molecular-Weight Allosteric Agonist of the Thyroid-Stimulating Hormone Receptor on Basal and Thyroliberin-Stimulated Activity of Thyroid System in Diabetic Rats
Source: Int J Mol Sci. 2025 Jan 15;26(2):703. doi: 10.3390/ijms26020703 (PMC11766125; doi:10.3390/ijms26020703)
Supplement: Supplementary file 1 [file ijms-26-00703-s001.zip › Figure S4.pdf]

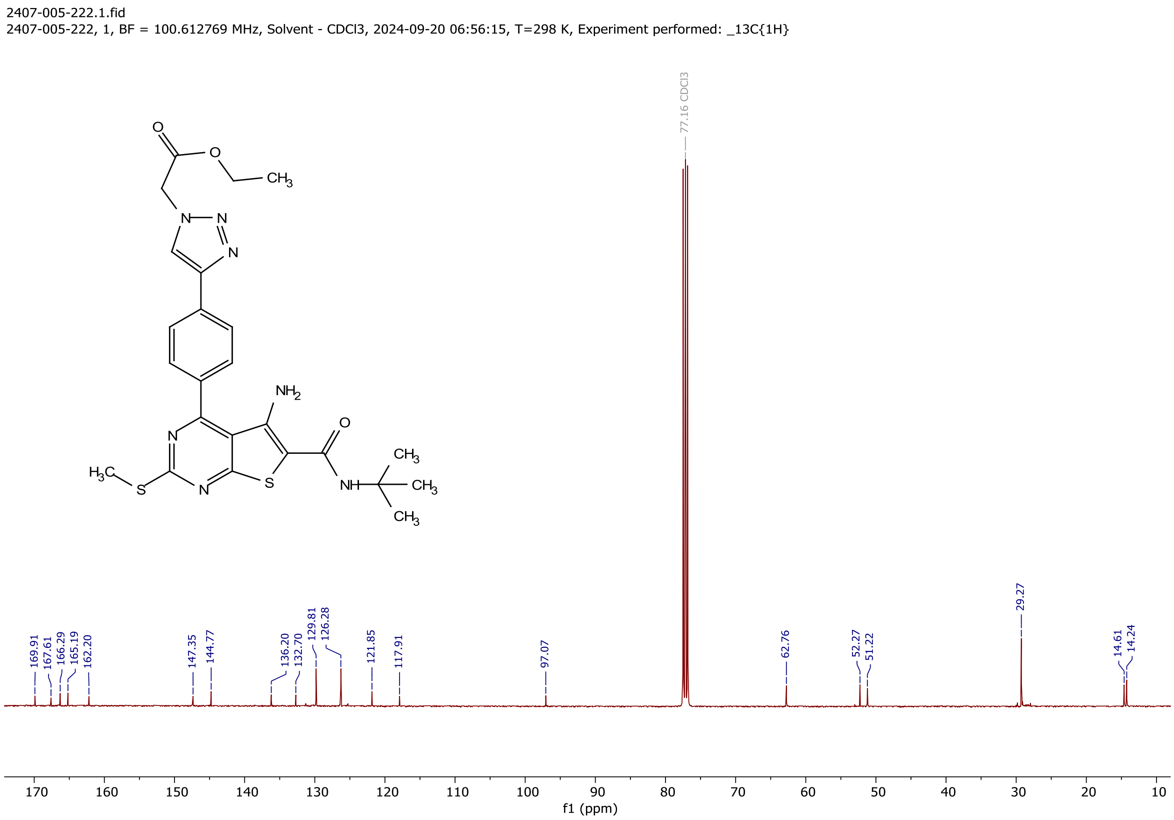

Figure S4. The 13C-NMR spectrum for the compound TPY3m, ethyl-2-(4-(4-(5-amino-6-(tert-butylcarbamoyl)-2-(methylthio)thieno[2,3-d]pyrimidin-4-yl)phenyl)-1H-1,2,3-triazol-1-yl) acetate.
