# Supplementary material for: Effect of a Low-Molecular-Weight Allosteric Agonist of the Thyroid-Stimulating Hormone Receptor on Basal and Thyroliberin-Stimulated Activity of Thyroid System in Diabetic Rats
Source: Int J Mol Sci. 2025 Jan 15;26(2):703. doi: 10.3390/ijms26020703 (PMC11766125; doi:10.3390/ijms26020703)
Supplement: Supplementary file 1 [file ijms-26-00703-s001.zip › Figure S5.pdf]

# MASS SPECTRUM REPORT

| | |
|---|---|
| Data Filename : VS_1.lcd | Instrument Name: Nexera_LCMS-9030 |
| Acquired | 28/04/2024 09:24 ESI |
| Interface : | 3,0 L/min |
| Nebulizing Gas Flow : | On |
| Heating Gas : | 10,0 L/min |
| Heating Gas Flow : | On |
| Interface heater : | 180 C |
| Interface Temperature : | 261 C |
| Desolvation Temperature : | ESI |
| Sub-Interface Ionization Mode : | MS |
| Start of Data Acquisition, Diverter Valve : | No Change |
| After Data Acquisition, Diverter Valve : | On |
| Drying Gas : | 10,0 L/min |
| Drying Gas Flow : | 250 C |
| DL Temperature : | 400 C |
| Heat Block : | |

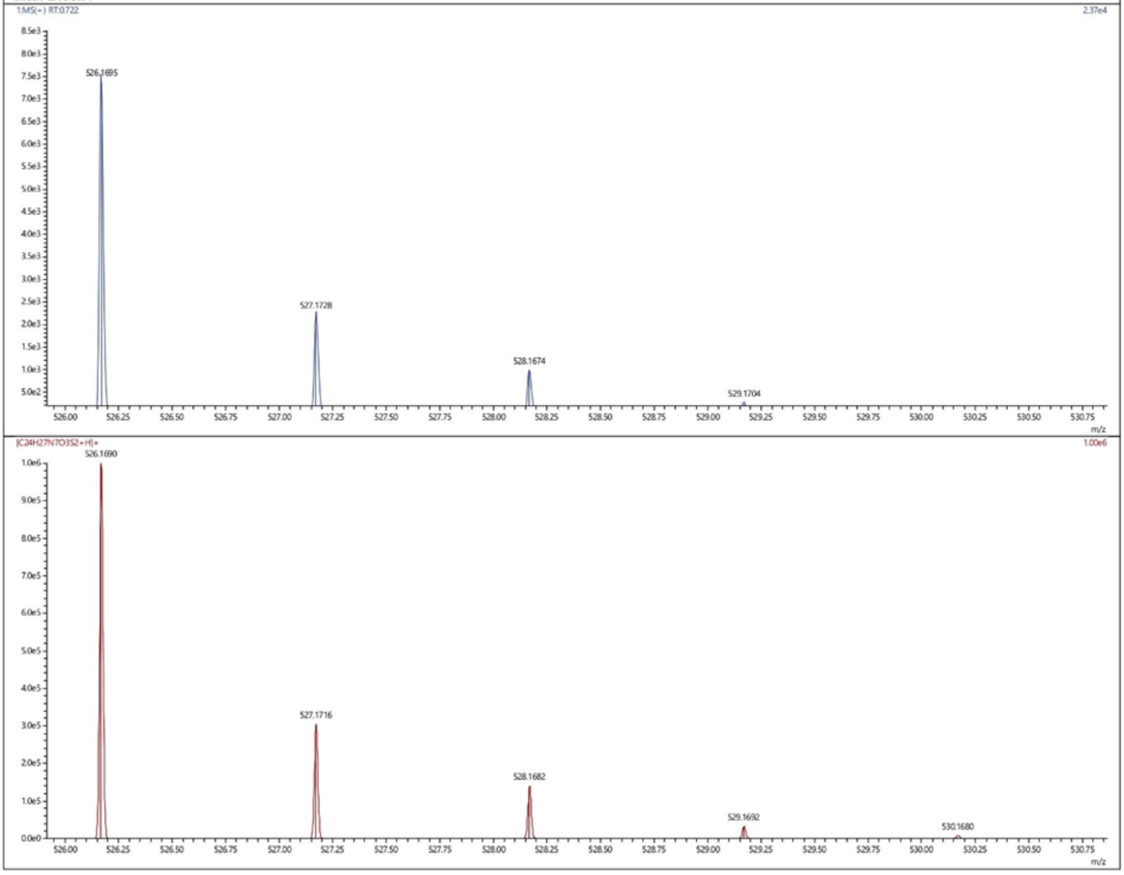


**Figure S5.** The mass spectrum for the compound TPY3m, ethyl-2-(4-(4-(5-amino-6-(*tert*-butylcarbamoyl)-2-(methylthio)thieno[2,3-d]pyrimidin-4-yl)phenyl)-1*H*-1,2,3-triazol-1-yl) acetate.
