# Supplementary material for: Effect of a Low-Molecular-Weight Allosteric Agonist of the Thyroid-Stimulating Hormone Receptor on Basal and Thyroliberin-Stimulated Activity of Thyroid System in Diabetic Rats
Source: Int J Mol Sci. 2025 Jan 15;26(2):703. doi: 10.3390/ijms26020703 (PMC11766125; doi:10.3390/ijms26020703)
Supplement: Supplementary file 1 [file ijms-26-00703-s001.zip › Table S1.pdf]

Table S1. Effect of treatment with TPY3m (single dose 20 mg/kg, i.p.) on blood testosterone levels in healthy male rats

**Hormone**	**C**	**C+TRH**	**C+TP**	**C+TP+TRH**
Testosterone, pmol/L	12.7 ± 1.6	10.5 ± 1.8	9.6 ± 1.1	12.9 ± 1.7
