# Supplementary material for: Effect of a Low-Molecular-Weight Allosteric Agonist of the Thyroid-Stimulating Hormone Receptor on Basal and Thyroliberin-Stimulated Activity of Thyroid System in Diabetic Rats
Source: Int J Mol Sci. 2025 Jan 15;26(2):703. doi: 10.3390/ijms26020703 (PMC11766125; doi:10.3390/ijms26020703)
Supplement: Supplementary file 1 [file ijms-26-00703-s001.zip › Table S2.pdf]

Table S2. The primers for the study of target and reference genes in the thyroid gland, pituitary and hypothalamus of rats using RT-PCR analysis

Gene	Forward/Reverse Sequence	AT	GenBank
Tshr	For: CTCGGACAAGACATGAGCCC	56	NM_012888.1
	Rev: GGTCCAGGGACTTGCTCAAA		
Tg	For: GCCCTAACTCATCCGTCCA	54	NM_030988.1
	Rev: TGTTGATAAGCCCATCGTCCT		
Tpo	For: TTGGATCTGGCATCACTGAACTT	56	NM_019353.2
	Rev: ATCTTGTTGACCATGCTTCTGTTG		
Nis	For: AAGTGACCGGGTTGGACATC	56	NM_052983.2
	Rev: AGCCAACGAGCATTACCACA		
Dio2	For: CGTCATCCTCAAGTGTCCCC	56	NM_031720.5
	Rev: TGGTACGCGCACATTACCTT		
Dio3	For: GCCCGTTGGTGCTCAATTTT	55	NM_017210.4
	Rev: CTGTGGGATGACGTAGGGTG		
Tsh-beta	For: TTGTGGGCAAGTGTCATCGT	56	NM_013116.2
	Rev: GCAGTAGGCACACTCTCTCC		
Trhr1	For: CCAAGCTAGCTCATAGGCCC	55	NM_013047.3
	Rev: GCATGCAAGTCAACAGGGTG		
pro-TRH	For: GAAAGCAAAGCACACACAGAGA	56	XM_039107125.1
	Rev: AGGACTCATTCCAGGCCAAC		
18S rRNA	For: GGACACGGACAGGATTGACA	56	NR_046237.1
	Rev: ACCCACGGAATCGAGAAAGA		
Actb	For: CTGGCACCACACCTTCTACA	56	NM_031144.3
	Rev: AGGTCTCAAACATGATCTGGGT		

Note. AT – annealing temperature (°C).
